# The influence of high glucose and high insulin on mechanisms controlling cell cycle progression and arrest in mouse C2C12 myoblasts: the comparison with IGF-I effect

**DOI:** 10.1007/s40618-013-0007-z

**Published:** 2014-01-09

**Authors:** K. Grabiec, M. Gajewska, M. Milewska, M. Błaszczyk, K. Grzelkowska-Kowalczyk

**Affiliations:** Department of Physiological Sciences, Faculty of Veterinary Medicine, Warsaw University of Life Sciences (SGGW), Nowoursynowska 159, 02-776 Warsaw, Poland

**Keywords:** G1cyclins, High glucose and insulin, IGF-I, Myogenesis

## Abstract

**Background:**

Myogenesis is susceptible to the availability of nutrients and humoral factors and suboptimal fetal environments affect the number of myofibers and muscle mass.

**Aim:**

We examined the mechanisms regulating cell cycle progression and arrest in skeletal myoblasts.

**Materials and methods:**

Mouse C2C12 myoblasts were subjected to proliferation or induction of differentiation in the presence of high glucose and high insulin (HGHI glucose 15 mmol/l, insulin 50 nmol/l), and these effects were compared with the influence of anabolic factor for skeletal muscle, insulin-like growth factor-I (IGF-I 30 nmol/l).

**Results:**

High glucose and high insulin, similarly to IGF-I, increased the intracellular level of cyclin A, cyclin B1 and cyclin D1 during myoblast proliferation. In HGHI-treated myoblasts, these cyclins were localized mostly in the nuclei, and the level of cdk4-bound cyclin D1 was augmented. HGHI significantly stimulated the expression of cyclin D3, total level of p21 and cdk-bound fraction of p21 in differentiating cells. The cellular level of MyoD was augmented by HGHI both in proliferating and differentiating myogenic cells.

**Conclusions:**

High glucose and insulin modify the mechanisms controlling cell cycle progression and the onset of myogenesis by: (1) increase of cyclin A, cyclin B1 and cyclin D1 in myoblast nuclei, and stimulation of cyclin D1-cdk4 binding; (2) increase in cyclin D3 and MyoD levels, and the p21-cdk4 complexes after induction of differentiation. Hyperglycemia/hyperinsulinemia during fetal or postnatal life could exert effects similar to IGF-I and can be, therefore, favourable for skeletal muscle growth and regeneration.

## Introduction

Skeletal muscle, a key insulin-responsive organ, composes 40–50 % of body mass, makes it the most important tissue for glucose and fatty acid utilization. The fetal stage is crucial for skeletal muscle development because, on general, there is no increase in muscle fiber numbers after birth [1]. Postnatal regenerative myogenesis supported by satellite cells present in mature muscle recapitulates fetal myogenesis [2]. In humans and some animals, as sheep and swine, secondary myogenesis that mainly accounts for skeletal muscle mass is susceptible to the availability of nutrients and humoral factors, and thus suboptimal fetal environments decreased the number of myofibers [3, 4]. Disrupted fetal skeletal muscle development leading to low or high birth weight affects the whole body glucose and fatty acid metabolism [5] and predisposes offspring to diabetes and obesity later in life [6, 7]. For these reasons, the potential mechanisms of influence of humoral factors associated with obesity and diabetes on muscle development and growth receive much attention.

Skeletal muscle formation or myogenesis is a complex and highly regulated process that involves the proliferation of myoblasts, followed by morphological, biochemical, and molecular modifications, which result in the formation of multinucleated myotubes. Proliferation and differentiation are two excluding processes [8]. Proliferation occurs through series of events arranged in precise order, typical of cell cycle (G1, S, G2, M phases), whereas cells undergoing differentiation have to withdraw cell cycle in G1 phase and except in specific circumstances, are unable to reenter the cell cycle in response to growth factors [9]. In skeletal myoblasts, cyclin-dependent kinase (cdk) 4 and 6 are crucial for regulation of proliferation [10]. Constitutive expression of mutated cyclin D1 and cdk4 allowed to preserve high proliferation activity without compromising a differentiation potential in myogenic cells in culture [11]. Cyclin D1 has been described as an important target in pathways regulating proper cell cycle withdrawal during skeletal myogenesis [12]. Several studies have demonstrated that p21 and pRb (retinoblastoma) proteins regulate proliferation and differentiation of myoblasts. The G1/S cdk inhibitor p21 blocks cell cycle progression before S phase through inactivation of cdks activity. The expression of p21 is upregulated during myogenesis that is associated with permanent cell cycle arrest of muscle cells [13]. pRb activity is governed by its phosphorylation state, i.e., hypophosphorylated pRb binds and sequesters transcription factors, most notably those of the E2F family, inhibiting the transcription of genes required to G1/S transition [14]. This cell cycle inhibitory function is abrogated when pRb undergoes phosphorylation mediated by cdk [15]. Cyclin D3 expression induces high levels of the cdk inhibitor p21, the increase in levels of myogenic genes such as MyoD, Myf5, and myogenin at an early stage during differentiation [16]. In differentiating myoblasts high levels of cyclin D3 occur, this protein forms complexes with not phosphorylated form of pRb protein [17] and inhibits the activity of cyclins determining cell cycle progression in differentiating myogenic cells [18].

Our recent observations performed on C2C12 cells revealed some modifications of the myogenic differentiation under high glucose and high insulin (HGHI) treatment manifested by an increased fusion index, myogenin and myosin heavy chain (MHC) expression on the 3rd day of myogenesis [19]. The purpose of the present study was to examine the cellular levels and localization of cyclins stimulating cell cycle progression (cyclin A, B1, D1), proteins essential for cell cycle arrest and the onset of myogenesis (cyclin D3, p21, pRb and MyoD), as well as the distribution of cyclin D1 and p21 in the cdk4 complexes in mouse C2C12 myoblasts exposed to HGHI. Effects of experimental factor in C2C12 myogenic cells were compared with the action of insulin-like growth factor-I, a potent anabolic factor of particular interest which is critical in promoting both proliferation and differentiation of skeletal muscle cells, muscle hypertrophy and muscle regeneration following injury [20].

## Materials and methods

### Cell culture

Research work, in in vitro system was carried out on the murine myogenic C2C12 cell line that undergoes proliferation and differentiation in response to growth factors present in the extracellular environment, thus being a useful model to study the mechanisms controlling the myogenesis.

C2C12 mouse myoblast cell line (satellite cells from thigh muscle) purchased from the European Collection of Animal Cell Culture (ECACC) was used for the study. Cell cultures free of contamination were maintained in an exponential phase of growth in 10 % FBS/DMEM, together with antibiotic–antimycotic mixture (Life Technologies), in controlled humidified air supplemented with 5 % CO_2_, at 37 °C. The growing medium was changed every other day. Proliferating myoblasts, after reaching ~40 % confluence, were subjected to 48-h exposure to experimental factor: high glucose (final concentration 15 mmol/l) in combination with high insulin (final concentration 50 nmol/l), added to 2 %FBS/DMEM. Medium containing experimental factors was prepared directly before using by resolving the appropriate amount of powdered glucose in DMEM, subsequent filtration and supplementation with FBS and antibiotic solutions, and addition of aliquot of sterile 1000 × concentrated working solution of insulin. Final concentration of insulin used in our study was 500 times higher than physiological concentration of this hormone in normal fed mice [21]. In our early study [22], 6-day differentiation of C2C12 myoblasts under these concentrations of glucose and insulin resulted in insulin and IGF-I resistance, which was manifested by the abolished insulin- or IGF-I-stimulated protein synthesis and impaired phosphorylation of protein kinase B, p70 s6k, mitogen-activated protein kinase and p90 rsk. Myogenic differentiation was induced when cells reached ~80 % confluence, by switching to the medium containing 2 % horse serum (HS) supplemented with high glucose in combination with high insulin, as described above. In order to compare potential effects of HGHI with potent anabolic factor for skeletal muscle, the separate set of experiments was performed with IGF-I (final concentration 30 nmol/l) added to the medium. This concentration of IGF-I was closed to the physiological concentration of this growth factor in normal fed mice [21] and, according to our recent study was effective in stimulating of myogenesis in C2C12 myoblasts, manifested by increased fusion index and the expression of myogenic regulatory factors: MyoD, myogenin and MHC [23]. The experiments with HGHI and IGF-I were performed in the identical conditions. Proliferating and differentiating control cultures were maintained in 2 % FBS/DMEM or 2 % HS/DMEM medium, respectively, containing 5 mmol/l glucose and traces of insulin derived from the addition of serum. To preserve the characteristics of the C2C12 cell line, the splitting of cells was done up to a maximum of seven times. Each experiment was conducted three times in triplicate.

### Assessment of cell viability and proliferation

Viability of proliferating and differentiating cells was determined using 3-(4,5-dimethylthiazol-2-yl)-2,5-diphenyltetrazolium bromide (MTT) assay. The cells were seeded in 96-well plates and after different incubation time, 180 μL of MTT solution (0.5 mg/ml) in PBS was added to each well. The plates were then incubated for 4 h at 37 °C. The reaction product, i.e., the formazan precipitates were then solubilized in 100 % DMSO (100 μl/well).

The crystal violet assay was performed to determine the total amount of nuclear DNA (cell proliferation). The cells cultured in 96-well plates were fixed with 75 and 100 % methanol for 20 min, and then monolayers were stained using crystal violet solution (2 mg/ml) for 5 min. The excess of unbound dye was removed by washing the plates with water. The bound crystal violet was released by adding 1 % SDS for 30 min. In both assays, the absorbance was measured on a multi-detection microplate reader Infinite 200 PRO Tecan™ (TECAN, Mannedorf, Switzerland) at a wavelength of 570 nm.

### Immunoblotting

Whole cell lysates were obtained using RIPA buffer supplemented with protease and phosphatase inhibitor cocktail (Sigma–Aldrich, St. Louis, MO, USA). Protein concentration in the lysates was determined using BCA kit according to the manufacturer’s instructions. Aliquots of cell extracts corresponding to 100 μg of protein were resolved by SDS-PAGE and then transferred onto PVDF membrane. The membranes were blocked with 5 % nonfat dry milk in TBS buffer and then incubated with appropriate primary antibodies (all supplied by Santa Cruz Biotechnology), followed by three 15-min washes in TBS containing 0.5 % Tween 20 (TBST), and 1-h incubation with secondary antibodies (at 1:5,000 dilution). The secondary antibodies were conjugated with appropriate IR fluorophores: IRDye^®^ 680 or IRDye^®^ 800 CW (IR- longer-wavelength near-infrared), which enable detection of the specific proteins directly on the PVDF membrane using Odyssey Infrared Imaging System (LI-COR Biosciences). The detection is based on the signal form the near-infrared (NIR) fluorophores-conjugated antibodies. Scan resolution of the instrument was set at 169 μm, and the intensity at 4, in standard protocol. Quantification of the integrated optical density (IOD = optical density × area) was performed with the analysis software provided with the Odyssey scanner (LI-COR Biosciences). The optical density of the band of each studied protein was presented in arbitrary units. This semi-quantitative method allowed to compare the level of protein of interest between the control and experimental treatments. Membranes were also reprobed with anti-actin antibody, to ensure that all lines contain equal amounts of total protein.

### Immunoprecipitation

To assess the potential protein interaction and protein redistribution in the complexes, the stage of immunoprecipitation previous to immunoblotting was used. Whole cell lysates containing ~300 μg of total protein were subjected to 12-h incubation with primary antibody for protein of interest previously adsorbed on the Protein A/G Plus agarose (Santa Cruz Biotechnology). Antigen–antibody complexes adsorbed on agarose beds were recovered by centrifugation, washed three times with PBS, and then subjected to SDS-PAGE, the electrotransfer and blotting with appropriate antibody, as was described above. The interactions of examined proteins were evaluated on the basis: (1) presence of cyclin D1 in the material after immunoprecipitation with the antibody specific for cdk4, and (2) presence of p21 protein in the material after immunoprecipitation with the antibody specific for cdk4 protein. The control probing with the antibody used for immunoprecipitation was also performed, to ensure that equal amounts of protein were precipitated and recovered from whole cell lysate.

### Immunofluorescence staining and confocal microscopy

Cell cultures were carried out directly on glass Lab-tec coverslips (Nunc Inc., USA). The cells were fixed with 3.7 % paraformaldehyde for 20 min at room temperature and permeabilized with 0.05 % Triton X-100 in PBS. The cells were incubated overnight in darkness at 4 °C with primary antibody anti-cyclin A, anti-cyclin B1, anti-cyclin D1, anti-p21, anti-pRb, anti-MyoD (all purchased from Santa Cruz Biotechnology) diluted 1:100 in PBS. The slides were then rinsed three times with PBS and incubated for 1 h with Alexa Fluor 488 secondary antibodies (Eugene, USA) diluted 1:500 in PBS. For nuclear visualization, cells were stained with 7-aminoactinomycin D (7-AAD, 5 μg/ml) in PBS for 15 min at room temperature. After rinsing, the coverslips were mounted on glass slides using Fluorview mounting medium (Sigma–Aldrich) and the cells were visualized by confocal laser scanning microscope FV-500 system (Olympus Optical Co, Hamburg, Germany). The combinations of excitation/emission were Argon 488 nm laser with 505–525 nm filter, for Alexa Fluor 488 and HeNe 543 nm laser with 610 nm filter for 7AAD nucleus staining. Stack of cross-sections were gathered separately for each fluorescence channel. Ten independent fields were acquired from each repetition of control and experimentally treated cell cultures and the images representative for each group were presented. The IOD values of green fluorescence corresponding to cytoplasmic localization of examined proteins, as well as a yellow light resulted from simultaneous excitation of red (7AAD) and green (specific antibody) fluorescence, manifesting nuclear translocation of proteins were evaluated using MicroImage analysis system (Olympus Optical Poland). The data were presented as total fluorescence of fields with similar cell density and as a nucleus/cytoplasm immunofluorescence ratio.

### Statistical analyses

The results of MTT and CV tests are representative of four separate experiments, whereas the data obtained by immunoblotting and immunofluorescence represent three separate experiments, performed in triplicate. Student’s *t* test was used for the comparison of two means (control vs each experimental treatment effect). In order to compare the effect of HGHI and IGF-I, the results were also evaluated using analysis of variance (ANOVA) in which three repetitions were nested within the experiment. In analysis of immunoblotting, results actin data were introduced as a constant co-variable. As the results in separate experiments gave a similar pattern of changes, all data within each group were combined to calculate a mean value ± SD. The analyses were performend using SPSS 12.0PL for Windows (SPSS Inc. & SPSS Poland).

## Results

**Fig. 1:**
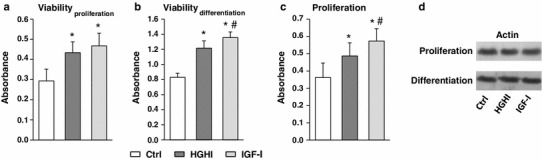
Effect of high glucose and high insulin combination (HGHI glucose concentration 15 mmol/l insulin concentration 50 nmol/l) and insulin-like growth factor-I (IGF-I, concentration 30 nmol/l) on cell viability assessed in MTT test (**a**, **b**), cell proliferation assessed in CV test (**c**) and the cellular content of actin (**d**) in C2C12 myogenic cell cultures. Data from MTT and CV assays represent mean ± SD, with *n* = 12/treatment condition. Blots are representative of three separate experiments. *Asterisk* indicates significantly different vs. control (Ctrl) for the same parameter. *Hash symbol* indicates significantly different vs. high glucose and high insulin effect for the same parameter

In order to verify the effect of HGHI on cell viability and proliferation, the MTT (a marker of mitochondrial respiration) and crystal violet (a marker of DNA content) assay, respectively, were employed. Proliferating C2C12 myoblasts exposed to HGHI for 48 h exhibited significantly higher cell respiration than control cultures (by 48 %, *p* < 0.001, Fig. 1a). Treatment of myoblasts with IGF-I (30 nmol/l) for 48 h markedly augmented cell respiration in comparison to control cultures (by 59 %, *p* < 0.001), but the growth factor effect did not differ significantly from HGHI-stimulated cell respiration (*p* > 0.05). Three days incubation of differentiating C2C12 cells with HGHI also augmented cell respiration (by 46 % vs control value, *p* < 0.001, Fig. 1b). Exposure of differentiating C2C12 cells to IGF-I augmented cell respiration (by 63 % vs control value, *p* < 0.001), which was also slightly but significantly higher than HGHI effect (*p* < 0.001). HGHI significantly stimulated myoblast proliferation assessed after 48-h exposure (by 34.5 % in comparison to control value, *p* < 0.01, Fig. 1c). IGF-I markedly stimulated myoblast proliferation assessed after 48-h treatment in comparison to control (by 58 %, *p* < 0.001), as well as to HGHI-treated cultures (*p* < 0.05). HGHI and IGF-I treatment did not alter the cellular level of actin, either in myoblasts or after induction of differentiation (Fig. 1d).

**Fig. 2:**
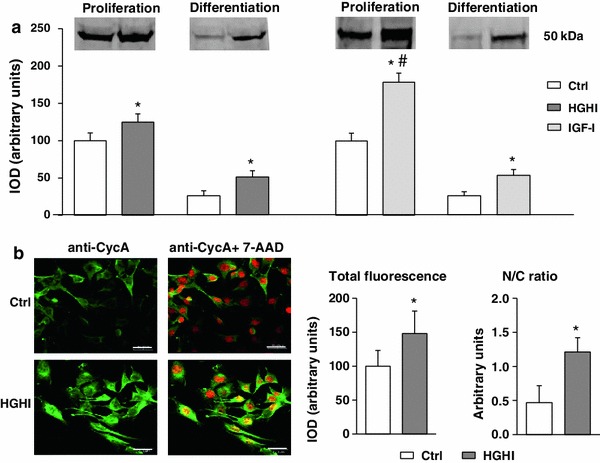
**a** Effect of high glucose and high insulin combination (HGHI glucose concentration 15 mmol/l insulin concentration 50 nmol/l) and insulin-like growth factor-I (IGF-I, concentration 30 nmol/l) on cyclin A (Cyc A) in C2C12 myogenic cell cultures. The level of Cyc A in whole cell lysates of proliferating myoblasts (“Proliferation”) or in 72 h after induction of myogenesis (“Differentiation”) was evaluated by immunoblotting. The densitometric quantitation of the specific bands (IOD, integrated optical density) is presented in arbitrary units with the value obtained in appropriate control (Ctrl) proliferating cells set as 100 %. As three separate experiments gave a similar pattern of results, all data within each group were combined to calculate mean ± SD, with *n* = 9/treatment conditions, and representative blots were presented. *Asterisk* indicates significantly different vs. appropriate control (Ctrl) for the same parameter and the same culture conditions. *Hash symbol* indicates significantly different vs. high glucose and high insulin effect for the same parameter. **b** Cellular content and localization of cyclin A in mouse C2C12 myoblasts proliferating for 24 h in 2 % FBS/DMEM (Control, Ctrl) or in the presence of HGHI. Cell cultures were stained with antibodies against Cyc A (*green*) and simultaneous nuclear staining with 7-AAD (*red*) was performed. Images are representative of ten independent fields in three separate experiments. *Bar* 20 μm. The IOD values of Cyc A-related green fluorescence were evaluated using MicroImage analysis system (Olympus Optical Poland). The data were presented as mean ± SD, with *n* = 10/treatment conditions of total fluorescence of fields with similar cell density and as a nucleus/cytoplasm immunofluorescence ratio. *Asterisk* indicates significantly different vs. control (Ctrl) value

As shown in Fig. 2a, HGHI markedly increased the intracellular level of cyclin A during myoblast proliferation. After induction of myoblast differentiation, the cellular content of cyclin A decreased dramatically but was still higher under HGHI treatment than in control cultures (*p* < 0.05). Exposition to IGF-I gave the similar pattern of results, except that the effect of growth factor on CycA in proliferating cells was significantly higher than in high glucose- and insulin-treated cultures. In control proliferating myoblasts, cyclin A was present both in the cytoplasm and in the nuclei (Fig. 2b). HGHI supplementation caused a marked increase in the level of cyclin A in a single cell as well as in cell number exhibiting a high cyclin A expression. Moreover, in HGHI-treated myoblasts, cyclin A-related green fluorescence overlapped with nuclear red 7-AAD fluorescence, which was manifested by yellow fluorescence (resulting from simultaneous excitation of green and red fluorochromes). This latter indicates that in the presence of HGHI, cyclin A was localized mainly in myoblast nuclei.

**Fig. 3:**
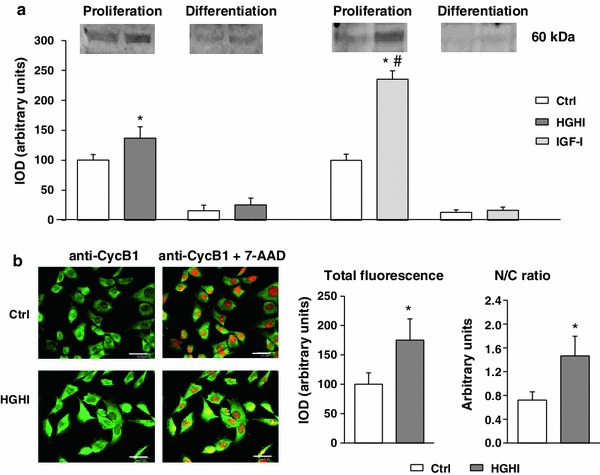
**a** Effect of high glucose and high insulin combination (HGHI glucose concentration 15 mmol/l insulin concentration 50 nmol/l) and insulin-like growth factor-I (IGF-I, concentration 30 nmol/l) on cyclin B1 (Cyc B1) in C2C12 myogenic cell cultures. The level of Cyc B1 in whole cell lysates of proliferating myoblasts (“Proliferation”) or in 72 h after induction of myogenesis (“Differentiation”) was evaluated by immunoblotting. The densitometric quantitation of the specific bands (IOD, integrated optical density) is presented in arbitrary units with the value obtained in appropriate control (Ctrl) proliferating cells set as 100 %. As three separate experiments gave a similar pattern of results, all data within each group were combined to calculate mean ± SD, with *n* = 9/treatment conditions, and representative blots were presented. *Asterisk* indicates significantly different vs. appropriate control (Ctrl) for the same parameter and the same culture conditions. *Hash symbol* indicates significantly different vs. high glucose and high insulin effect for the same parameter. **b** Cellular content and localization of cyclin B1 in mouse C2C12 myoblasts proliferating for 24 h in 2 % FBS/DMEM (Control, Ctrl) or in the presence of HGHI. Cell cultures were stained with antibodies against Cyc B1 (*green*) and simultaneous nuclear staining with 7-AAD (*red*) was performed. Images are representative of ten independent fields in three separate experiments. *Bar* 20 μm. The IOD values of Cyc B1-related green fluorescence were evaluated using MicroImage analysis system (Olympus Optical Poland). The data were presented as mean ± SD, with *n* = 10/treatment conditions of total fluorescence of fields with similar cell density and as a nucleus/cytoplasm immunofluorescence ratio. *Asterisk* indicates significantly different vs. control (Ctrl) value

Glucose and insulin also augmented the level of cyclin B1, associated with mitosis, in proliferating myoblasts, as assessed by immunoblotting (Fig. 3a). As expected, after induction of myoblast differentiation the cellular content of cyclin B1 decreased and was negligible both in control and in experimentally treated cultures. Like in the case of CycA, the effect of IGF-I on cyclin B1 in proliferating cells was more evident than the HGHI response. In myoblasts under control conditions, cyclin B1 was localized both in the cytoplasm and in the nuclei that was manifested by the appearance of yellow fluorescence, resulting from simultaneous excitation of green fluorescence attributed to cyclin B1 and red fluorescence of 7-AAD (Fig. 3b). High glucose and high insulin exposure led to the increase in number of cells exhibiting high level of cyclin B1 and nuclear cyclin B1 localization.

**Fig. 4:**
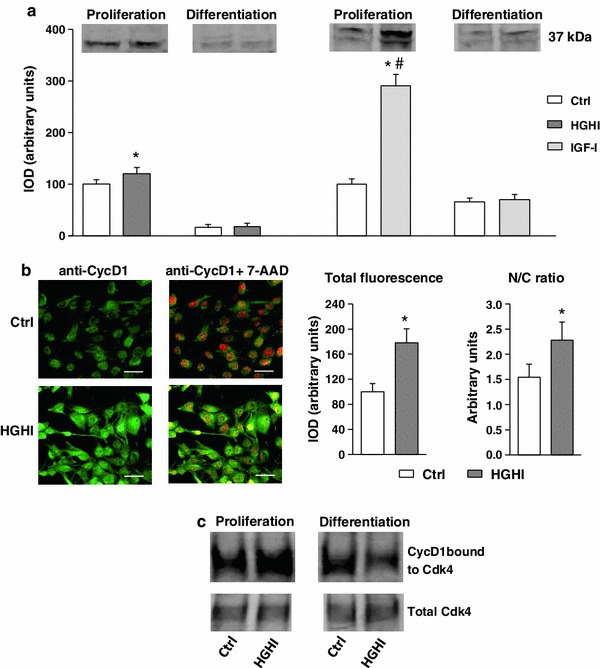
**a** Effect of high glucose and high insulin combination (HGHI glucose concentration 15 mmol/l insulin concentration 50 nmol/l) and insulin-like growth factor-I (IGF-I, concentration 30 nmol/l) on cyclin D1 (Cyc D1) in C2C12 myogenic cell cultures. The level of Cyc D1 in whole cell lysates of proliferating myoblasts (“Proliferation”) or in 72 h after induction of myogenesis (“Differentiation”) was evaluated by immunoblotting. The densitometric quantitation of the specific bands (IOD, integrated optical density) is presented in arbitrary units with the value obtained in appropriate control (Ctrl) proliferating cells set as 100 %. As three separate experiments gave a similar pattern of results, all data within each group were combined to calculate mean ± SD, with *n* = 9/treatment conditions, and representative blots were presented. *Asterisk* indicates significantly different vs. appropriate control (Ctrl) for the same parameter and the same culture conditions. *Hash symbol* indicates significantly different vs. high glucose and high insulin effect for the same parameter. **b** Cellular content and localization of cyclin D1 in mouse C2C12 myoblasts proliferating for 24 h in 2 % FBS/DMEM (Control, Ctrl) or in the presence of HGHI. Cell cultures were stained with antibodies against Cyc D1 (*green*) and simultaneous nuclear staining with 7-AAD (*red*) was performed. Images are representative of ten independent fields in three separate experiments. *Bar* 20 μm. The IOD values of Cyc D1-related green fluorescence were evaluated using MicroImage analysis system (Olympus Optical Poland). The data were presented as mean ± SD with *n* = 10/treatment conditions of total fluorescence of fields with similar cell density and as a nucleus/cytoplasm immunofluorescence ratio. *Asterisk* indicates significantly different vs. control (Ctrl) value. **c** The level of cyclin D1 bound to cyclin-dependent kinase 4 (Cdk4) in proliferating and differentiating myogenic cells. The presence of Cyc D1-cdk4 complexes was visualized by immunoprecipitation with the antibody specific for cdk4. The control probing with the antibody used for immunoprecipitation was also performed to ensure that equal amounts of protein were precipitated and recovered from whole cell lysate. Blots are representative of three separate experiments

In proliferating myoblasts treated with HGHI, an increased level of cyclin D1 assessed by immunoblotting was observed and, again, IGF-I appeared to be a more potent activator of cyclin expression (Fig. 4a). The cellular content of cyclin D1 in myogenic cells induced to differentiation was low and was not affected by experimental treatment. In control proliferating cultures, cyclin D1 was localized predominantly in myoblast nuclei (Fig. 4b). HGHI augmented the level of cyclin D1 in a single myoblast, and this increase concerned both the cytoplasm (strong green fluorescence) and the nuclei (yellow fluorescence). HGHI increased the level of cyclin D1 bound in complexes with cdk4 in proliferating C2C12 myoblasts, whereas after induction of differentiation cyclin D1 associated with cdk4 fell under experimental treatment (Fig. 4c).

**Fig. 5:**
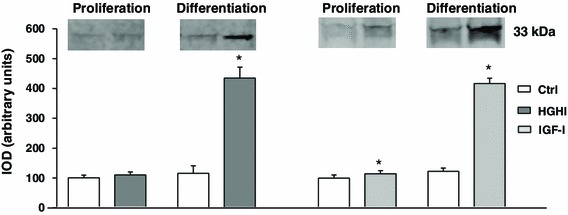
Effect of high glucose and high insulin combination (HGHI, glucose concentration 15 mmol/l insulin concentration 50 nmol/l) and insulin-like growth factor-I (IGF-I, concentration 30 nmol/l) on cyclin D3 in C2C12 myogenic cell cultures. The level of Cyc D3 in whole cell lysates of proliferating myoblasts (“Proliferation”) or in 72 h after induction of myogenesis (“Differentiation”) was evaluated by immunoblotting. The densitometric quantitation of the specific bands (IOD integrated optical density) is presented in arbitrary units with the value obtained in appropriate control (Ctrl) proliferating cells set as 100 %. As three separate experiments gave a similar pattern of results, all data within each group were combined to calculate mean ± SD with *n* = 9/treatment conditions, and representative blots were presented. *Asterisk* indicates significantly different vs. control (Ctrl) for the same culture conditions

In cell cultures subjected to differentiation, both HGHI and IGF-I significantly stimulated the expression of cyclin D3 associated with the cell cycle exit and induction of myogenesis (Fig. 5). The cellular content of cyclin D3 was low and not modified by HGHI in proliferating myoblasts.

**Fig. 6:**
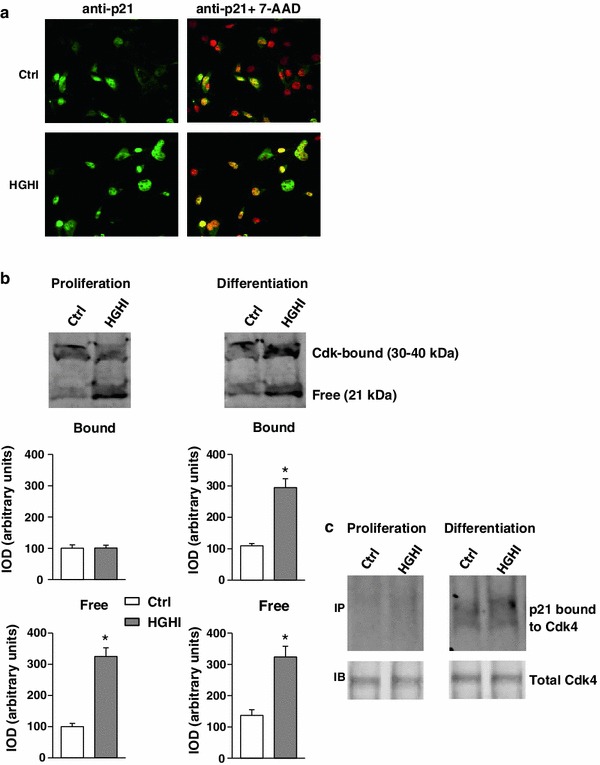
Effect of high glucose and high insulin combination (HGHI, glucose concentration 15 mmol/l insulin concentration 50 nmol/l) on p21 in C2C12 myogenic cell cultures. **a** Cellular content and localization of p21 in mouse C2C12 myoblasts proliferating for 24 h in 2 % FBS/DMEM (Control, Ctrl) or in the presence of HGHI. Cell cultures were stained with antibodies against p21 (*green*), and simultaneous nuclear staining with 7-AAD (*red*) was performed. Images are representative of ten independent fields in three separate experiments. *Bar* 20 μm. **b** The level of p21 in whole cell lysates of proliferating myoblasts (“Proliferation”) or in 72 h after induction of myogenesis (“Differentiation”) was evaluated by immunoblotting. The densitometric quantitation of the specific bands (IOD integrated optical density) is presented in arbitrary units with the value obtained for each isoform (Bound and Free) in control (Ctrl) proliferating cells set as 100 %. As three separate experiments gave a similar pattern of results, all data within each group were combined to calculate mean ± SD with *n* = 9/treatment conditions, and representative blots were presented. *Asterisk* indicates significantly different vs. control (Ctrl) for the same p21 isoform and the same culture conditions. **c** The level of p21 bound to cyclin-dependent kinase 4 (Cdk4) in proliferating and differentiating myogenic cells. The presence of p21-cdk4 complexes was visualized by immunoprecipitation with the antibody specific for cdk4. The control probing with the antibody used for immunoprecipitation was also performed, to ensure that equal amounts of protein was precipitated and recovered from whole cell lysate. Blots are representative of three separate experiments

As was shown in Fig. 6a, the cell cycle inhibitor p21 was present predominantly in myoblast nuclei both in control and experimental conditions. The immunoblotting analysis allowed to detect two forms of 21 protein: one with apparent molecular weight of 21 kDa, that corresponds to unbound fraction of this protein, and another one reflecting the amount of protein complexed with cdks. Under proliferation conditions, HGHI caused an increase in unbound p21 fraction, while the amount of cdk-bound p21 level was not grossly affected. After induction of myogenesis, the total level of p21 increased under glucose and insulin treatment, and this effect was attributed to the augmented cdk-bound fraction (Fig. 6b). In proliferating control and HGHI-treated myoblasts p21 bound to cdk4 was hardly detected, whereas in myogenic cells subjected to differentiation experimental treatment increases the fraction of p21 associated with cdk4 (Fig. 6c).

**Fig. 7:**
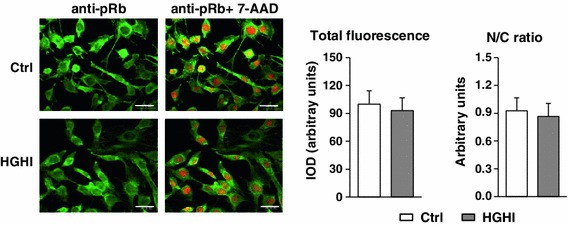
Effect of high glucose and high insulin combination (HGHI, glucose concentration 15 mmol/l insulin concentration 50 nmol/l) on cellular content and localization of pRb in mouse C2C12 myoblasts proliferating for 24 h in 2 % FBS/DMEM (Control, Ctrl) or in the presence of experimental factors. Cell cultures were stained with antibodies against pRb (*green*) and simultaneous nuclear staining with 7-AAD (*red*) was performed. Images are representative of ten independent fields in three separate experiments. *Bar* 20 μm. The IOD values of pRb-related green fluorescence were evaluated using MicroImage analysis system (Olympus Optical Poland). The data were presented as mean ± SD with *n* = 10/treatment conditions of total fluorescence of fields with similar cell density and as a nucleus/cytoplasm immunofluorescence ratio

Proliferating myogenic cells expressed pRb and HGHI did not augment significantly the cellular level of this protein assessed by immunofluorescence (Fig. 7). Similarly, the cellular pRb localization (i.e., nucleus vs cytoplasm) did not differ between two groups.

**Fig. 8:**
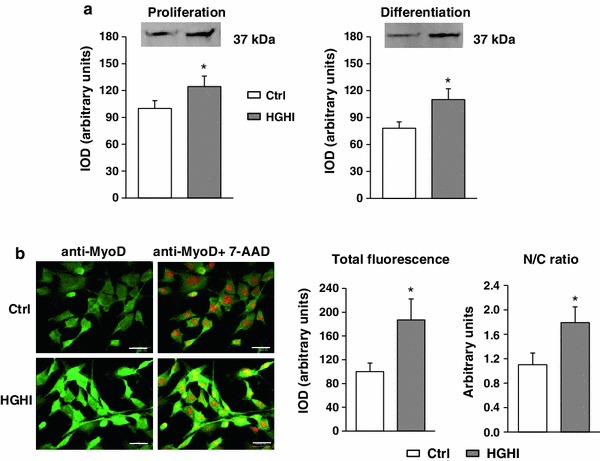
Effect of high glucose and high insulin combination (HGHI, glucose concentration 15 mmol/l insulin concentration 50 nmol/l) on MyoD in C2C12 myogenic cell cultures. **a** The level of MyoD in whole cell lysates of proliferating myoblasts (“Proliferation”) or in 72 h after induction of myogenesis (“Differentiation”) was evaluated by immunoblotting. The densitometric quantitation of the specific bands (IOD, integrated optical density) is presented in arbitrary units with the value obtained in control (Ctrl) proliferating cells set as 100 %. As three separate experiments gave a similar pattern of results, all data within each group were combined to calculate mean ± SD, with *n* = 9/treatment conditions, and representative blots were presented. **b** Cellular content and localization of MyoD in mouse C2C12 myoblasts proliferating for 24 h in 2 % FBS/DMEM (Control, Ctrl) or in the presence of HGHI. Cell cultures were stained with antibodies against MyoD (*green*), and simultaneous nuclear staining with 7-AAD (*red*) was performed. Images are representative of ten independent fields in three separate experiments. *Bar* 20 μm. The IOD values of MyoD-related green fluorescence were evaluated using MicroImage analysis system (Olympus Optical Poland). The data were presented as mean ± SD with *n* = 10/treatment conditions of total fluorescence of fields with similar cell density and as a nucleus/cytoplasm immunofluorescence ratio. *Asterisk* indicates significantly different vs. control (Ctrl) for the same parameter and the same culture conditions

In proliferating myoblasts and after induction of myogenesis the cellular level of MyoD, the early myogenic regulatory factor, was augmented by glucose and insulin treatment (Fig. 8a). Confocal microscopy revealed that in control proliferating C2C12 cultures, MyoD was present both in cytoplasm and in nuclei. Supplementation of growing medium with HGHI led to the increase in MyoD-associated immunofluorescence in both cellular compartments (Fig. 8b).

## Discussion

The number of muscle precursor cells is an important factor determining skeletal muscle mass. The mechanisms controlling myoblast proliferation, cell cycle exit and the onset of myogenesis as well as their potential modification by extracellular factors acting during fetal and postnatal period play an essential role in health status in later life and, thus, merit interest. The purpose of the present study was, therefore, to examine the regulation of the onset of muscle differentiation by high glucose combined with high insulin, one of humoral factors associated with insulin resistance and obesity. Currently, relatively little is known about the mechanisms controlling muscle differentiation under HGHI treatment. Taking into consideration that high levels of insulin may be a risk factor in carcinogenesis, acting probably by binding with IGF-I receptor and/or decreasing IGF binding proteins [24], one can expect a mitogenic action of insulin. Moreover, an induction of myoblast differentiation in vitro has been observed at high insulin supply [25]. However, recently Nedachi et al. [26] have found the ability of insulin myogenic action to be markedly affected by extracellular glucose, i.e., insulin exerted its myogenic effect only in the presence of high ambient glucose, whereas its effect was diminished under low glucose conditions.

In our study, a pattern of expression of cell cycle markers was consistent with previous reports, i.e., so-called G1 cyclins (cyclin A, B1 and D1) associated with cell cycle progression were present in proliferating myoblasts and dramatically fell after the induction of myogenesis; cyclin D3 and pRb were higher in differentiating cells, whereas cdk4 levels were similar in proliferating and differentiating cells [17, 27]. HGHI present in extracellular environment of proliferating myoblasts augmented the cellular level of cyclins that play an essential role in cell cycle progression. The increase in cyclin A, which promotes cell cycle progression mainly in S and G2 phases, in cultures treated with HGHI resulted from both increased expression in a single myoblast and increased number of myoblasts expressing cyclin A. Noteworthy, in glucose- and insulin-treated myoblasts cyclin A was present mainly in the nuclei that allows to predict its activity. Experimental treatment also augmented the cellular level of cyclin B1, associated with mitosis, both in the cytoplasm and in myoblast nuclei. In myoblasts subjected to proliferation under HGHI the increased level of cyclin D1, particularly in the nucleus, was observed. Previous work has established cyclin D1 as a predominant cyclin that controls the rate of progression through the G1 phase of the cell cycle [28]. Moreover, virtually all the cyclin D1 activity was attributable to cdk4 in cultured mouse fibroblasts and muscle cells [18]. In our present study, cyclin D1 was bound with cdk4, and exposition of myoblasts to HGHI augmented this fraction of cyclin D1. Taken together, our results indicate that under HGHI treatment, the stimulation of the expression and activity of cyclins promoting cell cycle progression takes place (Figs. 2, 3, 4) and this is consistent with the increase in myoblast proliferation, assessed by crystal violet assay (Fig. 1c). It should be noticed that the expression of cyclins associated with cell cycle progression was not completely compromised after induction of differentiation. Moreover, in differentiating myogenic cells treated with HGHI, the cellular levels of cyclin A, B1 and D1 were higher than in control cultures. The potential explanation of this phenomenon is that, under conditions promoting myogenesis, the mitogenic effect of HGHI persists and is sufficient to stimulate cell cycle progression in a limited cell population that did not trigger the differentiation program. Alternatively, HGHI activates the expression of cyclins and their functions, which are independent of cell cycle regulation. In fact, the roles of cyclins and cyclin regulatory proteins in transcriptional regulation, cell migration, cytoskeletal dynamics and apoptosis have already been described [9, 29]. The potential contribution of G1 cyclins as regulators of these processes and their importance in myogenesis under HGHI treatment requires further investigation.

The expression of p21 is upregulated during myogenesis leading to cdks inactivation and permanent cell cycle arrest [30]. Indeed, an immunoblotting analysis of p21 revealed not only the band of ~21 kDa reflecting an unbound form of the protein, but also the band with apparent higher molecular weight, representing p21 probably bound in complexes with cdks. It has been early described that the role of p21 in cell cycle regulation depends on cellular content of this protein: at low concentrations, p21 is required for forming active complexes with cdks and for the stimulation of cell cycle progression, whereas an inhibition of cell cycle appears at higher p21 levels [31]. Further studies confirm this hypothesis and revealed that p21 is important for the assembly and nuclear import of cyclinD1/cdk4 [32]. Keeping in mind the dual role of p21 in cell cycle regulation one could suppose that in myoblast cultures treated with HGHI cell cycle is not inhibited by p21, since the increase in p21 level concerns an unbound fraction of this protein (lower band on Fig. 6b). According to our immunoprecipitation results (Fig. 6c) in proliferating myoblasts p21 bound to cdk4 was hardly detected, which is in contrast with the blot presented in Fig. 6b, where the bands manifesting p21 bound to cdks (upper band) were visible. The potential explanation of this apparent discrepancy is the presence of cdk other than cdk4 in complexes with p21. These cdks (i.e., cdk1, cdk2 or cdk6), all displaying molecular weight of ~30–40 kDa, could be bound to and inactivated by p21, especially in control cultures maintained in medium partly serum-depleted (2 % FBS/DMEM). An addition of HGHI to the medium can therefore play a role of mitogenic signals sufficient to release cdks from p21-bound complexes, which results in activation of cell proliferation. Under differentiation conditions, cdk4 seems to be an important target for p21. The increase in p21 bound to and inactivating cdk4 (Fig. 6c), the augmented level of cyclin D3 (Fig. 5) antagonizing the activity of other G1 cyclins, as well as the rise in MyoD (Fig. 8) could be favourable for the onset of myogenesis under HGHI.

The mitogenic effect of HGHI observed in our study appears to be independent of pRb. We found similar levels of this protein under control and HGHI treatment, assessed by immunofluorescence and confocal microscopy. Moreover, the cellular localization of pRb (nucleus vs. cytoplasm) was similar in control and experimentally treated myoblasts (Fig. 7). The potential explanation of this phenomenon is that the activity of cell cycle promoting cyclins, presumably increased under HGHI treatment, is not sufficient to cause the changes in pRb protein turnover to be detected in our study. It cannot be excluded that the level and/or activity of pRb is not a limiting factor in the mechanisms mediating the mitogenic effect of HGHI. The other signaling elements serving as potential downstream substrates for cell cycle promoting cyclins that transmit HGHI-exerted effects on cell cycle regulation should be investigated.

IGF-I, a potent growth factor, stimulated the levels of the proteins promoting myoblast cell cycle progression (Figs. 2, 3, 4) in agreement with a marked mitogenic effect (Fig. 1). According to early observations, IGF-I treatment has been shown to upregulate the expression of cdk4 and cyclin D1 genes and increase Rb protein phosphorylation in rat L6E9 cells [reviewed in 33]. In more recent study, however, IGF-I did not augment the proliferation of satellite cells, suggesting that IGF-I-dependent skeletal muscle hypertrophy results from an increase in number of cells engaged in differentiation, probably decreasing the pule of reserve cells [34]. The apparent discrepancy between such an observation and our study can be attributed to the characteristic of experimental models, particularly to the difference in proliferation potential of isolated satellite cells and C2C12 myoblast cell line. Regarding cyclins promoting cell cycle progression (A, B1 and D1) IGF-I appeared to be a more potent stimulator than HGHI, but it is unlikely to have biological significance due to general qualitative and quantitative similarity of these effects.

Increasing data from human and animal study have shown that maternal glucose intolerance and diabetes increase the risk of delivery of larger and fatter infants [summarized in 7, 35] with this latter being attributed to enhanced adipose tissue mass. On the other hand, skeletal muscles grow by an increase in the number of cells (i.e., hyperplasia) that occurs mainly during fetal life, and the total number of muscle fibers is set by the end of gestation [36]. The skeletal muscle mass is, therefore, closely related to the number of cells that trigger myogenic program and are able to undergo hypertrophy at later stages of muscle development. In this regard, our present observations suggest that macrosomia in offspring resulting from the exposure to high maternal glucose and insulin concentrations may involve the excessive growth of skeletal muscle, due to increased number of myoblasts contributing to the formation of muscle fibers. We concluded that high concentrations of glucose and insulin modify the activity of the mechanisms controlling both cell cycle progression and the onset of myogenesis. This effect was manifested by: (1) increase of cyclin A, cyclin B1 and cyclin D1 in myoblast nuclei, and stimulation of cyclin D1-cdk4 binding; (2) increase in cyclin D3 and MyoD levels, and the p21-cdk4 complexes after induction of differentiation. Hyperglycemia/hyperinsulinemia during fetal or postnatal life could exert effects similar to IGF-I and can be, therefore, favourable for skeletal muscle growth and regeneration.

## Abbreviations

Cdk: Cyclin-dependent kinase
CV: Crystal violet
DMEM: Dulbecco’s modified Eagle’s medium
FBS: Fetal bovine serum
HS: Horse serum
IOD: Integrated optical density
MTT: 3-(4,5-dimethylthiazol-2-yl)-2,5-diphenyltetrazolium
MyoD: Myogenic determination factor
pRb: Retinoblastoma protein
RIPA: RadioImmunoPrecipitation Assay
SDS-PAGE: Sodium dodecylsulphate polyacrylamide gel electrophoresis
